# Supplementary material for: A new sports garment with elastomeric technology optimizes physiological, mechanical, and psychological acute responses to pushing upper-limb resistance exercises
Source: PeerJ. 2024 Mar 6;12:e17008. doi: 10.7717/peerj.17008 (PMC10924454; doi:10.7717/peerj.17008)
Supplement: Supplemental Information 2 — ICC: intraclass correlation coefficient, interpreted as: poor (< 0.50), moderate (0.50 – 0.75), good (0.75 – 0.90), and excellent (> 0.90); CV: coefficient of variation, interpreted as excellent (≤ 10%), good (10 – 20%), acceptable (20 – 30%), and poor (> 30%); RMS: root mean square as a measure of the neuromuscular activity. [file peerj-12-17008-s002.docx]

| **Table S1: Intersession reliability of the physiological variables.** | | | | | |
| --- | --- | --- | --- | --- | --- |
| **Variable** | **Exercise** | **Elastomeric garment** | | **Placebo garment** | |
|  |  | **ICC** | **CV (%)** | **ICC** | **CV (%)** |
| RMS of the pectoralis major | Seated shoulder press | 0.99 | 6.29 | 0.98 | 6.71 |
|  | Push-ups | 0.97 | 7.19 | 0.99 | 4.94 |
| RMS of the triceps brachii | Seated shoulder press | 0.98 | 5.07 | 0.99 | 5.91 |
|  | Push-ups | 0.99 | 4.02 | 0.98 | 7.81 |
| RMS of the anterior deltoid | Seated shoulder press | 0.98 | 4.85 | 0.99 | 3.84 |
|  | Push-ups | 0.99 | 3.60 | 0.99 | 4.10 |
| RMS of the rectus abdominis | Seated shoulder press | 0.99 | 6.00 | 0.99 | 3.48 |
|  | Push-ups | 0.93 | 2.54 | 0.99 | 2.29 |
| Blood lactate | Seated shoulder press | 0.84 | 12.31 | 0.74 | 15.14 |
|  | Push-ups | 0.90 | 11.42 | 0.97 | 6.47 |
| Heart rate | Seated shoulder press | 0.95 | 9.45 | 0.97 | 7.41 |
|  | Push-ups | 0.93 | 5.75 | 0.82 | 6.81 |
| Systolic blood pressure | Seated shoulder press | 0.73 | 3.85 | 0.83 | 4.81 |
|  | Push-ups | 0.92 | 4.15 | 0.92 | 6.50 |
| Diastolic blood pressure | Seated shoulder press | 0.73 | 3.57 | 0.89 | 7.67 |
|  | Push-ups | 0.78 | 7.60 | 0.66 | 8.35 |

ICC: intraclass correlation coefficient, interpreted as: poor (< 0.50), moderate (0.50 – 0.75), good (0.75 – 0.90), and excellent (> 0.90); CV: coefficient of variation, interpreted as excellent (≤ 10%), good (10 – 20%), acceptable (20 – 30%), and poor (> 30%); RMS: root mean square as a measure of the neuromuscular activity.
