# Supplementary material for: A new sports garment with elastomeric technology optimizes physiological, mechanical, and psychological acute responses to pushing upper-limb resistance exercises
Source: PeerJ. 2024 Mar 6;12:e17008. doi: 10.7717/peerj.17008 (PMC10924454; doi:10.7717/peerj.17008)
Supplement: Supplemental Information 3 — ICC: intraclass correlation coefficient, interpreted as: poor (< 0.50), moderate (0.50 – 0.75), good (0.75 – 0.90), and excellent (> 0.90); CV: coefficient of variation, interpreted as excellent (≤ 10%), good (10 – 20%), acceptable (20 – 30%), and poor (> 30%); 1stMPV: mean propulsive velocity of the first repetition; PMVP: peak mean propulsive velocity of the set; AMVP: average of the mean propulsive velocities of the set; 1stRPE, rating perceived exertion of the first repetition. [file peerj-12-17008-s003.docx]

| **Table S2: Intersession reliability of the mechanical and psychological variables.** | | | | | |
| --- | --- | --- | --- | --- | --- |
| **Variable** | **Exercise** | **Elastomeric garment** | | **Placebo garment** | |
|  |  | **ICC** | **CV (%)** | **ICC** | **CV (%)** |
| 1^st^VMP | Seated shoulder press | 0.83 | 7.06 | 0.93 | 5.11 |
|  | Push-ups | 0.91 | 4.70 | 0.76 | 8.54 |
| PMPV | Seated shoulder press | 0.85 | 6.84 | 0.74 | 6.86 |
|  | Push-ups | 0.94 | 3.79 | 0.89 | 6.28 |
| AMPV | Seated shoulder press | 0.76 | 6.76 | 0.64 | 4.92 |
|  | Push-ups | 0.95 | 4.45 | 0.57 | 10.53 |
| Repetitions | Seated shoulder press | 0.96 | 3.87 | 0.95 | 4.35 |
|  | Push-ups | 0.96 | 5.70 | 0.92 | 7.30 |
| 1^st^RPE | Seated shoulder press | 0.60 | 23.42 | 0.79 | 21.91 |
|  | Push-ups | 0.75 | 24.33 | 0.67 | 16.64 |

ICC: intraclass correlation coefficient, interpreted as: poor (< 0.50), moderate (0.50 – 0.75), good (0.75 – 0.90), and excellent (> 0.90); CV: coefficient of variation, interpreted as excellent (≤ 10%), good (10 – 20%), acceptable (20 – 30%), and poor (> 30%); 1^st^MPV: mean propulsive velocity of the first repetition; PMVP: peak mean propulsive velocity of the set; AMVP: average of the mean propulsive velocities of the set; 1^st^RPE, rating perceived exertion of the first repetition.
