# Supplementary material for: A new sports garment with elastomeric technology optimizes physiological, mechanical, and psychological acute responses to pushing upper-limb resistance exercises
Source: PeerJ. 2024 Mar 6;12:e17008. doi: 10.7717/peerj.17008 (PMC10924454; doi:10.7717/peerj.17008)
Supplement: Supplemental Information 4 [file peerj-12-17008-s004.docx]

**Abbreviations**

- RPE: Rate of perceived exertion
- 1REP: First repetition
- LASTREP: Last repetition
- PRESS: Seated shoulder press
- ELASTOMERIC: Sports garment with elastomeric technology
- PLACEBO: Placebo garment
- MPV: Mean propulsive velocity
- SBP: Systolic blood pressure
- DBP: Diastolic blood pressure
- HR: Heart rate
- RMS Root mean square
- RECTUSABD: Rectus abdominis
