# Supplementary figures and images for: A new sports garment with elastomeric technology optimizes physiological, mechanical, and psychological acute responses to pushing upper-limb resistance exercises
Source: PeerJ. 2024 Mar 6;12:e17008. doi: 10.7717/peerj.17008 (PMC10924454; doi:10.7717/peerj.17008)

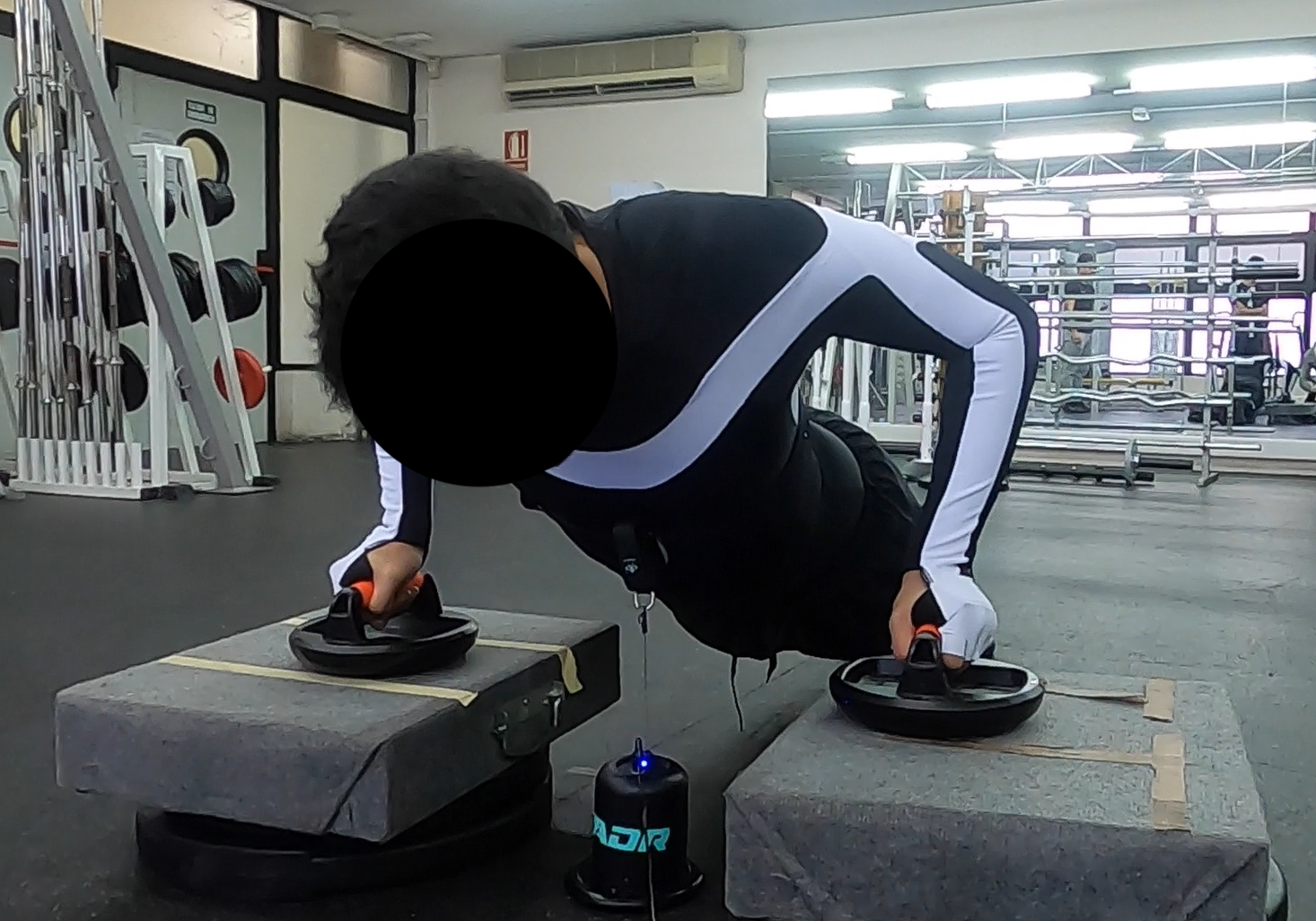

Supplement: Supplemental Information 5 [file peerj-12-17008-s005.zip › Supplementary Figure 1. Push-up 1.png]

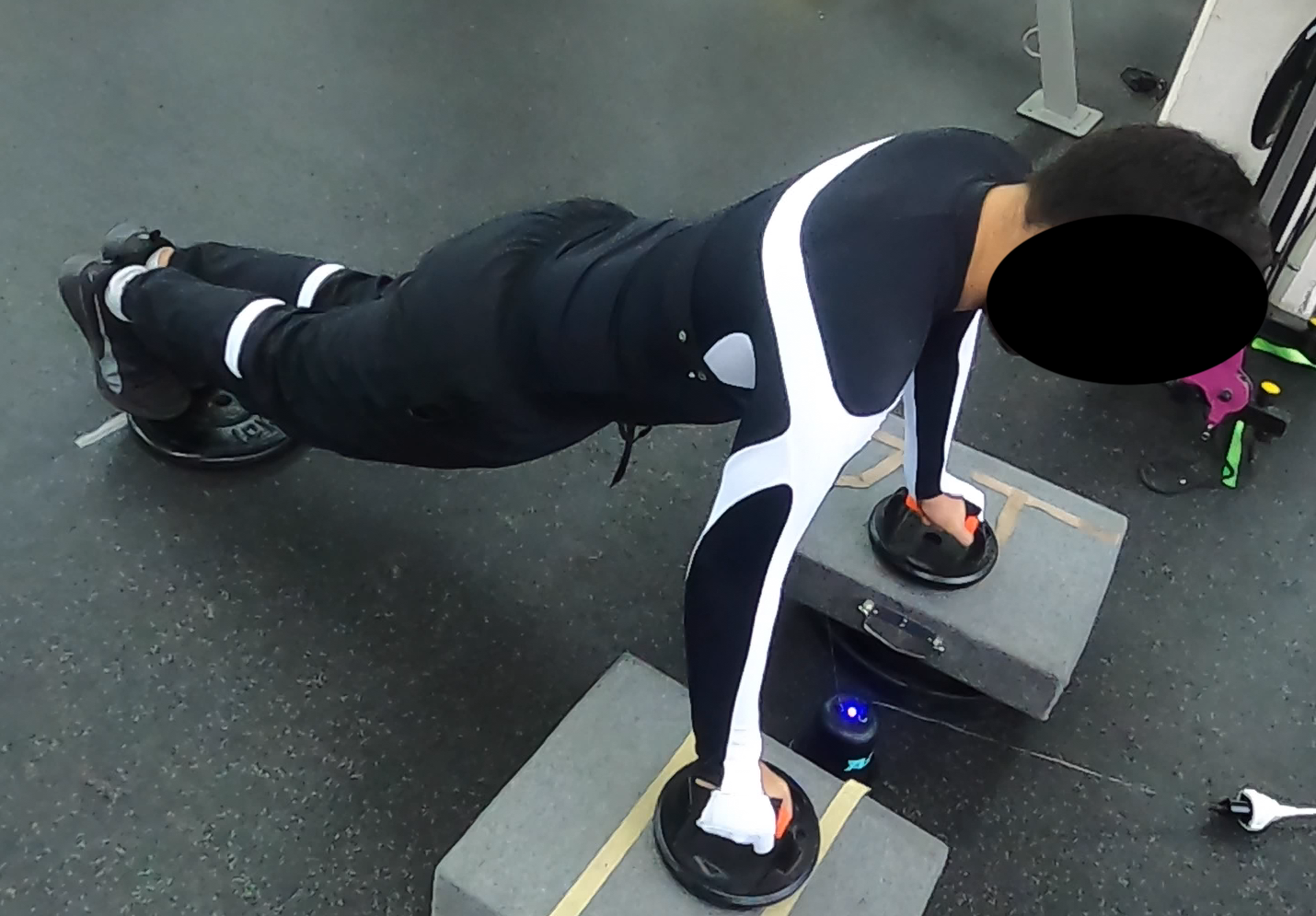

Supplement: Supplemental Information 5 [file peerj-12-17008-s005.zip › Supplementary Figure 2. Push-up 2.png]

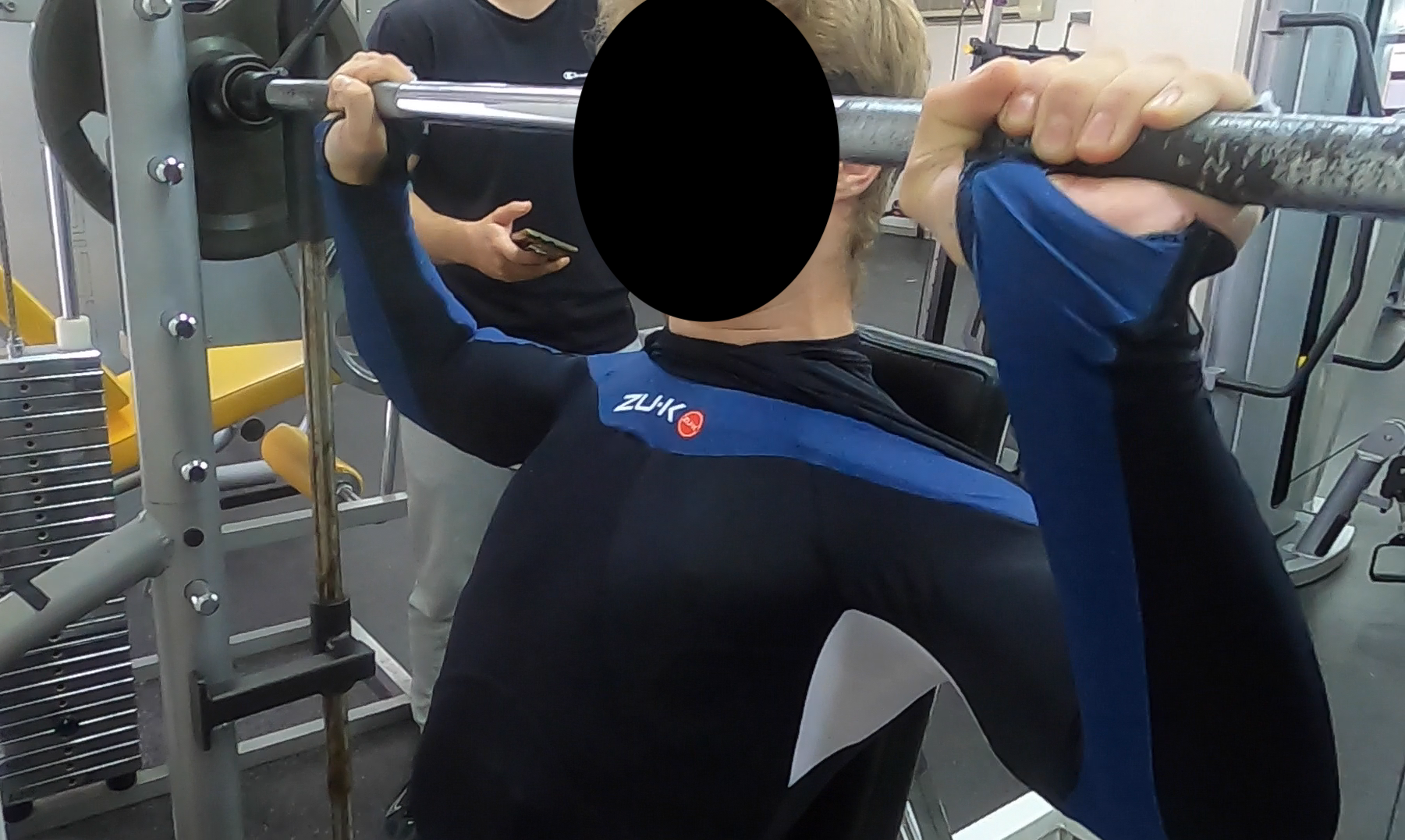

Supplement: Supplemental Information 5 [file peerj-12-17008-s005.zip › Supplementary Figure 3. Seated shoulder press 1.png]

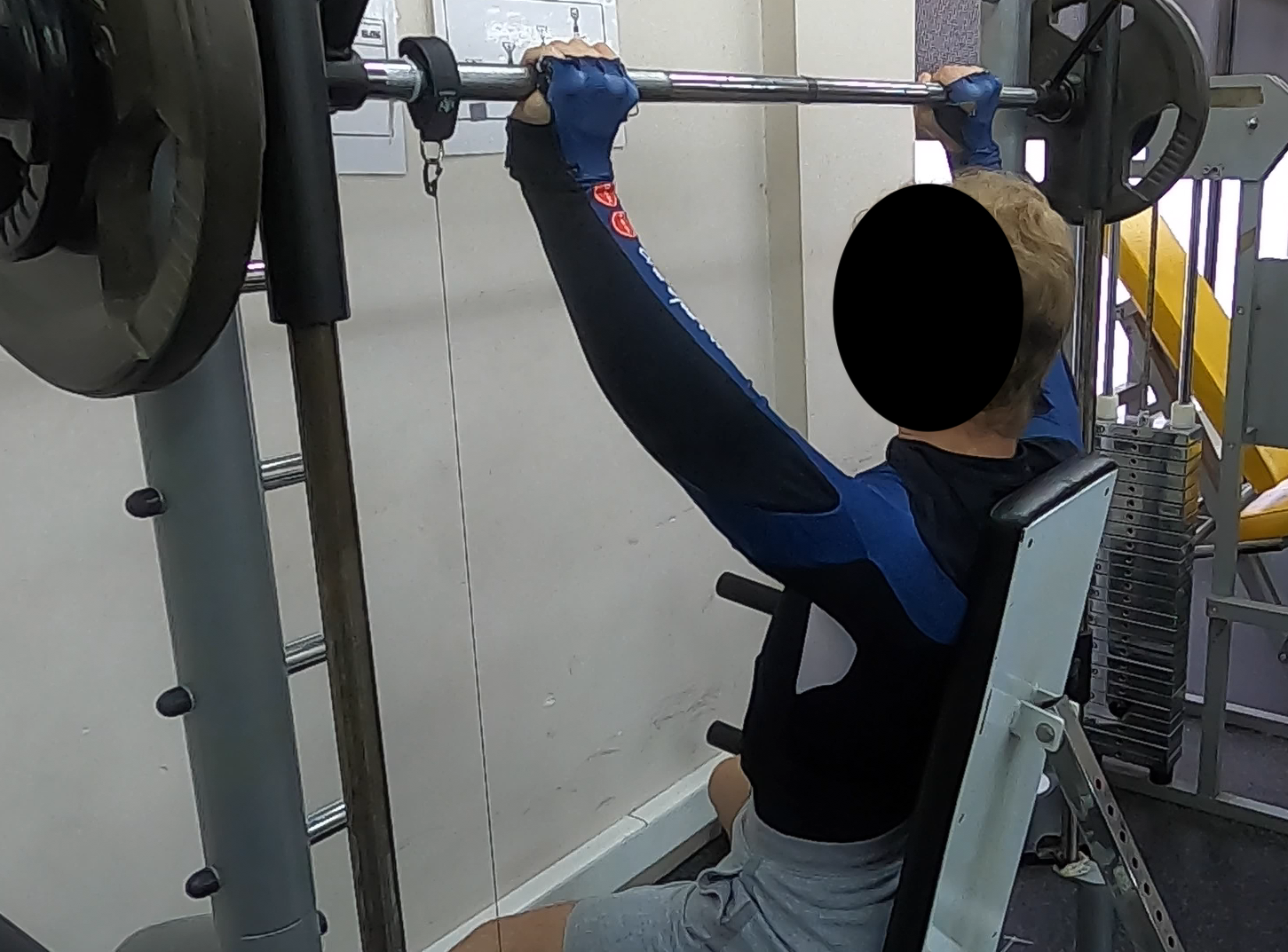

Supplement: Supplemental Information 5 [file peerj-12-17008-s005.zip › Supplementary Figure 4. Seated shoulder press 2.png]
